# Estimated power output for a distance run and maximal oxygen uptake in young adults

**DOI:** 10.3389/fphys.2023.1110802

**Published:** 2023-02-07

**Authors:** Gen-Min Lin, Kun-Zhe Tsai, Xuemei Sui, Carl J. Lavie

**Affiliations:** ^1^ Department of Internal Medicine, Hualien Armed Forces General Hospital, Hualien City, Taiwan; ^2^ Department of Medicine, Tri-Service General Hospital, National Defense Medical Center, Taipei, Taiwan; ^3^ Department of Stomatology of Periodontology, Mackay Memorial Hospital, Taipei, Taiwan; ^4^ Department of Exercise Science, Arnold School of Public Health, University of South Carolina, Columbia, SC, United States; ^5^ Ochsner Clinical School, John Ochsner Heart and Vascular Institute, The University of Queensland School of Medicine, New Orleans, LA, United States

**Keywords:** cardiopulmonary exercise testing, maximal oxygen uptake, estimated power output, run field test, velocity

## Abstract

**Background:** Both cardiopulmonary exercise testing (CPET) and run field tests are recommended by the American Heart Association for assessing the maximal oxygen uptake (VO_2_ max) of youth. Power output was highly correlated with VO_2_ max in CPET. However, it is unclear regarding the correlations of time and estimated power output (EPO) for a run field test with VO_2_ max obtained from CPET in young adults.

**Methods:** This study included 45 participants, aged 20–40 years, from a sample of 1,120 military personnel who completed a 3,000-m run field test in Taiwan in 2020. The participants subsequently received CPET using the Bruce protocol to assess VO_2_ max in the same year. According to the physics rule, EPO (watts) for the run field test was defined as the product of half body mass (kg) and [distance (3000-m)/time (s) for a run field test]. Pearson product–moment correlation analyses were performed.

**Results:** The Pearson correlation coefficient (r) of time against EPO for the run field test was estimated to be 0.708 (*p* <0.001). The correlation coefficient between the time for the run field test and VO_2_ max (L/min) in CPET was estimated to be 0.462 (*p* = 0.001). In contrast, the correlation coefficient between time for the run field test and VO_2_ max scaled to body mass in CPET was estimated to be 0.729 (*p* <0.001). The correlation coefficient of EPO for the run field test against VO_2_ max in CPET was estimated to be 0.813 (*p* <0.001).

**Conclusion:** In young adults, although the time for a run field test was a reliable estimate of VO_2_ max scaled to body mass, EPO proportional to the mean square velocity was found as a superior estimate of VO_2_ max.

## Introduction

Cardiorespiratory fitness (CRF) is inversely associated with the risk of metabolic syndrome, diabetes mellitus, cardiovascular diseases (CVD), and mortality in the general population (Carnethon et al., 2009; Mehta et al., 2020; Wang et al., 2021; Lin et al., 2022; Sui et al., 2022). Obtaining greater CRF levels for sedentary individuals has been proposed as one of the major preventive measures to reduce the severity and burden of atherosclerosis in developed countries (Lavie et al., 2019; Sanchis-Gomar et al., 2022). The gold standard for CRF assessment is maximal oxygen uptake (VO_2_ max) with or without an adjustment for body mass from cardiopulmonary exercise testing (CPET). The main advantages of CPET include a strictly controlled environment, e.g., a maintained indoor temperature, atmospheric pressure, and calibration gas. However, the limitation to CPET is facility-dependent, and examinees require a tolerance to wear a mask during the graded exercise testing. Therefore, CPET may not be feasible for some specific populations, such as children and the elderly, and might not be practical at a large population level.

Based on the findings of previous studies, the American Heart Association (AHA) has recommended VO_2_ max and some alternative measures for the CRF levels of youth (Mayorga-Vega et al., 2015; Raghuveer et al., 2020). For instance, the performance of a field-based 20-m shuttle run test or distance run test has a moderate-to-high correlation with VO_2_ max obtained from CPET, whereas the performance of a field-based step test or 6-minute walk test has only a low-to-moderate correlation with VO_2_ max in children or adolescents (Raghuveer et al., 2020).

In addition, previous studies also revealed that the peak power output of the heart or body measured in CPET has a high correlation with VO_2_ max in patients with recovering heart failure and athletes (Hawley and Noakes, 1992; Jakovljevic et al., 2011). Physiologically, most oxygen uptake is translated to energy output during peak exercise. However, to the best of our knowledge, there have been no studies with regard to the correlation between estimated power output (EPO) for a run field test and VO_2_ max in CPET. The correlation between a run test performance and VO_2_ max in CPET was not clarified in adults and varied by study (Cooper, 1968; O’Gorman et al., 2000; Casajus and Castagna, 2007). The aim of this study was to investigate the correlations of time and EPO for a distance run field test with VO_2_ max measured from CPET in a group of young military adults.

## Materials and methods

### Study population

The study included 45 participants, aged 20–40 years, without any medication use, randomly selected from the cardiorespiratory fitness and health in eastern armed forces (CHIEF) study participants (N = 1,120) (Lin et al., 2021; Liu et al., 2021) in 2020, which aimed to carry out a preliminary study on the correlation of time for a 3000-m run field test with VO_2_ max from CPET. All participants received exercise training daily, e.g., a 3,000-m run within 25 min in the morning at the military base for over 6 months. A history of moderate physical activity, such as a limited-time 3,000-m run in the morning per week in the past half year, was obtained from each participant. Each participant underwent the 2020 annual health examinations for physical examinations (Hsu et al., 2022) in the Hualien Armed Forces General Hospital of Taiwan. Each participant also underwent the 2020 annual military exercise test for a 3,000-m run field test to assess endurance capacity. Within 2 weeks of the 3,000-run field test, the 45 volunteers were scheduled for CPET to objectively assess VO_2_ max.

### Anthropometric and blood pressure (BP) measurements

Anthropometric parameters, i.e., body height and weight, were measured in a standing position by a medical staff member in the annual military exercise test. Body mass index was defined as the body weight divided by body height squared (kg/m^2^). Overweight or obesity was defined as a body mass index ≥27.5 kg/^2^ for Asians, according to the recommendations of the World Health Organization (Jih et al., 2014). The BP and pulse rate of each participant were automatically measured using the same device (FT201, Parama-Tech Co., Ltd., Fukuoka, Japan), which utilized the oscillometric method (Lin et al., 2016; Lin et al., 2020a; Lin et al., 2020b). The BP was measured once over the right arm in a sitting position after resting for longer than 15 min and was recorded by a medical staff member. If the pulse rate was ≥90 beats per minute, systolic BP ≥140 mmHg, or diastolic BP ≥90 mmHg was found, the participant would undergo another two rounds of hemodynamic parameter measurements, which were averaged as the final result.

### The 3000-m run field test

The 3,000-m run field test was performed outdoors on a flat playground of the Military Physical Training and Testing Center in Hualien, Taiwan, at 16:00. Each participant wore sweat suits and did not carry any additional objects. The whole running process of each participant was video recorded and supervised by eight military sports officers. The 3,000-m run field test was carried out if there was no heavy rain, and the coefficient of the heat stroke risk formula, the product of relative humidity (%) and outdoor temperature (Celsius scale) x 0.1, was less than 40. The time for the 3,000-m run field test was utilized to evaluate the endurance capacity of each participant. EPO for the run field test was defined as “1/2 x body mass (kg) x square of mean velocity (m/s),” on the basis of the physics rule of the kinetic energy theorem (Serway and Jewett, 2004). The mean velocity was calculated by the formula “distance (3,000-m) divided by time (s) for the run field test.”

### The performance of CPET

CPET was performed on a Trackmaster TMX-428 stress treadmill (SCHILLER, Baar, Switzerland) using the standard Bruce protocol. The same supervisor conducted all of the CPETs throughout the study. All participants were asked not to consume caffeine or alcohol for 12 h or longer before the CPET and exercised after a 2-h postprandial period. The room for the CPET used an air conditioning system to maintain a constant temperature of approximately 22 degrees Celsius. Throughout the CPET, electrocardiography and BP were monitored. The rates of oxygen uptake (VO_2_), production of carbon dioxide (VCO_2_), tidal volume (Vt), end-tidal partial pressure of carbon dioxide (PETCO_2_), and respiratory rate were recorded breath by breath using a Cardiovit CS-200 Excellence Ergo-Spiro analytic system (SCHILLER, Baar, Switzerland). VO_2_ max was defined as the average of VO_2_ during the last minute of maximal exercise.

### Statistical analysis

Characteristics of overall participants for a 3,000-m run field test and those for both a 3,000-m run field test and CPET were presented as numbers (%) for categorical variables and mean ± standard deviation for continuous variables, respectively. Continuous variables were compared using analysis of variance if the Kolmogorov–Smirnov test for the normal distribution was met; otherwise, the Wilcoxon signed-rank test was used. Categorical variables were compared using Fisher’s exact test. The Pearson product–moment correlation coefficient was used to determine the association strength of time and EPO for a 3,000-m run field test with VO_2_ max scaled to body mass or not in CPET. The correlations of time and EPO for a 3,000-m run field test were performed for a comparison between selected participants for both a 3,000-m run field test and CPET and a sample of age-, sex-, body mass index-, and BP-matched participants from the overall study participants. Scatter plots between time and EPO for the 3,000-m run field test and VO_2_ max scaled for body mass or not in CPET were obtained. Internal validation was performed for those whose body mass index <27.5 kg/m^2^. A value of *p* <0.05 was considered significant. All analyses were carried out using SPSS version 25.0 for Windows (IBM Corp., Armonk, NY, United States). This study has been approved by the Institutional Review Board of the Clinical Ethics Committee of the Mennonite Christian Hospital (No. 16-05-008), Hualien City, Eastern Taiwan, R.O.C., and written informed consent was obtained from all participants.

## Results

### Clinical characteristics of the participants

Table 1 shows the clinical characteristics of the participants for a 3,000-m run field test (N = 1,120) and those for both a 3,000-m run field test and CPET (N = 45). The characteristics of the selected participants for CPET were similar to the original overall sample, except greater age, pulse rate, and BP levels were observed in participants for CPET. The mean age of the participants for CPET was approximately 30 years old, and over 90% of the participants were males. Since only one woman was included for the CPET, the characteristics of men were also compared between the original group (N = 911) and the selected group (N = 44), and the results are provided in Supplementary Table S1, which show consistent findings.

**TABLE 1:** Clinical Characteristics of the Overall Participants for a Run Field Test and the Selected Participants for a Cardiopulmonary Exercise Testing.

	N = 45	N = 1120	*p*-value
Sex, males (%)	44 (97.8)	911 (81.3)	0.07
Age, years	29.93 ± 7.05	27.61 ± 5.87	0.01
Body height, cm	170.74 ± 6.47	170.80 ± 6.67	0.93
Body mass, kg	72.74 ± 11.81	71.93 ± 12.21	0.66
Body mass index, kg/m2	24.95 ± 3.86	24.58 ± 3.54	0.49
Pulse rate, beats per min	77.50 ± 11.18	67.09 ± 10.95	<0.001
Systolic blood pressure, mmHg	127.68 ± 11.83	116.97 ± 12.83	<0.001
Diastolic blood pressure, mmHg	80.32 ± 10.39	69.09 ± 9.88	<0.001
Time for a 3000-m run, secs	876.62 ± 94.34	893.55 ± 106.19	0.29
EPO for a 3000-m run, watts	437.44 ± 105.22	421.12 ± 114.18	0.34
Moderate activity per week			
100-150 minutes	8 (17.8)	224 (20.0)	0.92
150-300 minutes	18 (40.0)	423 (37.8)	
>300 minutes	19 (42.2)	473 (42.2)	

Abbreviation: EPO, estimated power output.

EPO was defined as 1/2 x body mass (kg) x (3000-m/time for a 3000-m run test)^2^.

### Correlations between time and EPO for a 3000-m run field test

**FIGURE 1:**
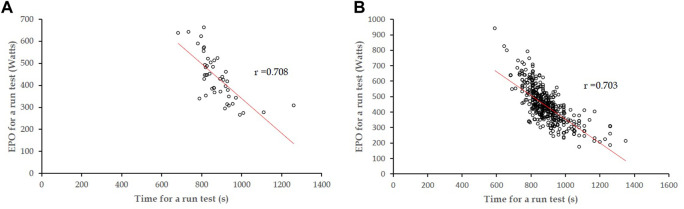
**(A)** Correlation coefficient (r) of time against EPO for a 3,000-m run field test, estimated to be 0.708 (*p* <0.001) in participants for both a 3,000-m run field test and CPET. **(B)** Correlation coefficient estimated to be 0.703 (*p* <0.001) in the variable-matched participants for a 3,000-m run field test.

The correlation coefficient (r) of time against EPO for a 3,000-m run field test was estimated to be 0.708 (*p* <0.001) in participants for both a 3,000-m run field test and CPET (Figure 1A), which was close to the correlation coefficient (r = 0.703 and *p* <0.001) in the age-, sex-, body mass index-, and BP-matched samples of 707 participants for a 3000-m run test (Figure 1B). The characteristics of the variable-matched population are provided in Supplementary Table S2. The correlation coefficients for men only were in line with the main findings, and the results are provided in Supplementary Figure S1.

### Correlations of time for a 3000-m run field test against VO_2_ max in CPET

**FIGURE 2:**
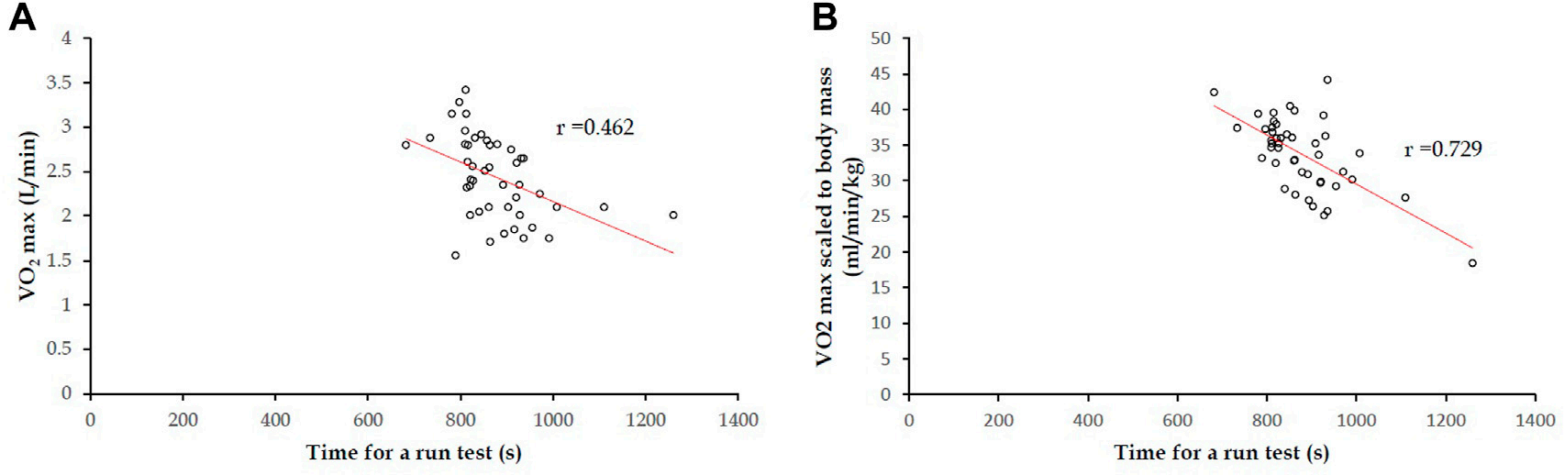
In 45 participants, for both a 3,000-m run field test and CPET: **(A)** correlation coefficient of time for a 3,000-m run field test against VO_2_ max (L/min) was 0.462 (*p* = 0.001); **(B)** correlation coefficient of time for a 3,000-m run field test with VO_2_ max scaled to body mass (kg) was 0.729 (*p* <0.001).

The correlation coefficient of time for a 3000-m run field test against VO_2_ max (L/min) in CPET was estimated to be 0.462 (*p* = 0.001) (Figure 2A). In contrast, the correlation coefficient between time for a 3000-m run field test and VO_2_ max scaled to body mass (kg) in CPET was estimated to be 0.729 (*p* <0.001) (Figure 2B). The correlation coefficients for men only were in line with the main findings, and the results are provided in Supplementary Figure S2.

## Correlations of EPO for a 3000-m run field test against VO_2_ max in CPET

**FIGURE 3:**
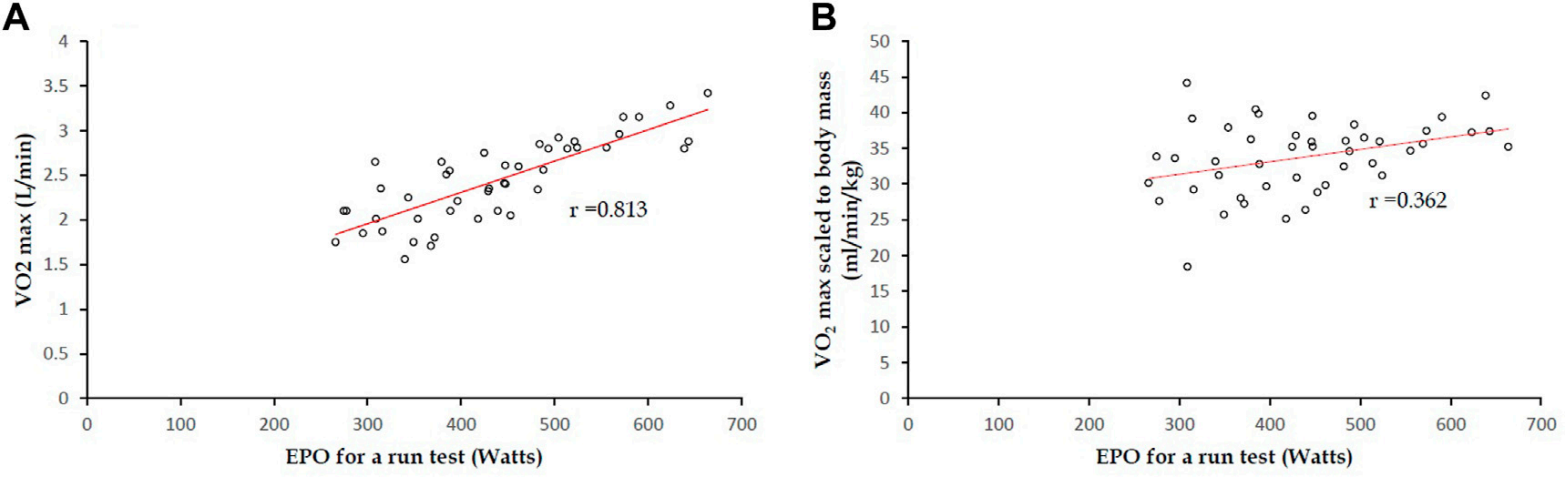
In 45 participants, for both a 3,000-m run field test and CPET: **(A)** correlation coefficient of EPO for a 3000-m run field test against VO_2_ max (L/min) was 0.813 (*p* <0.001); **(B)** correlation coefficient between EPO for a 3,000-m run field test and VO_2_ max scaled to body mass (kg) was estimated to be 0.364 (*p* <0.001).

The correlation coefficient of EPO for a 3,000-m run field test against VO_2_ max (L/min) in CPET was estimated to be 0.813 (*p* <0.001) (Figure 3A). However, the correlation coefficient between EPO for a 3,000-m run field test and VO_2_ max scaled to body mass (kg) in CPET was estimated to be only 0.364 (*p* <0.001) (Figure 3B). The correlation coefficients for men only were in line with the main findings, and the results are provided in Supplementary Figure S3.

### Internal validation for non-obese participants

**FIGURE 4:**
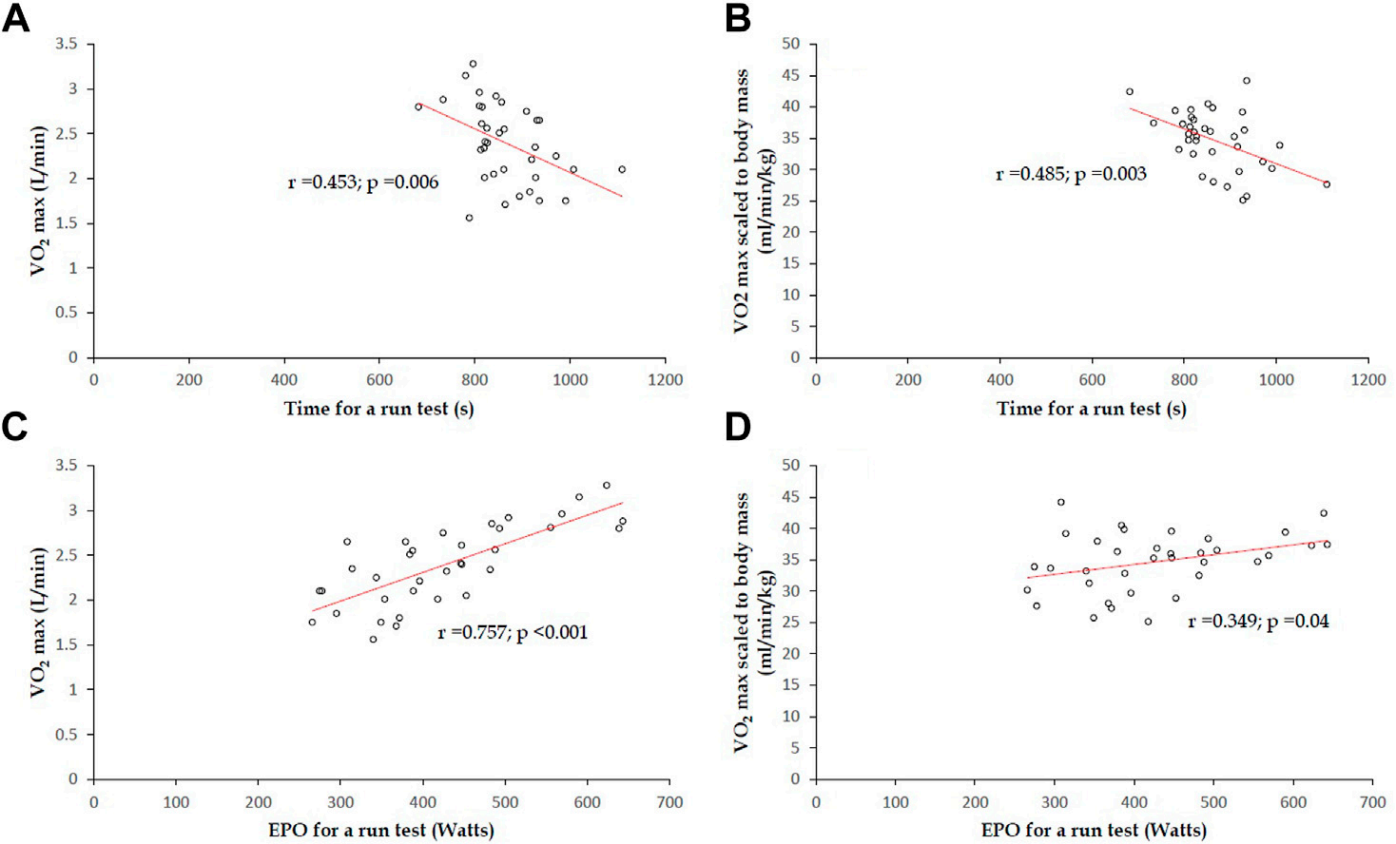
In 35 participants with body mass index <27.5 kg/m^2^, for both a 3000-m run field test and CPET: **(A)** correlation coefficient of time for a 3000-m run field test against VO_2_ max (L/min) was estimated to be 0.453 (*p* = 0.006); **(B)** correlation coefficient of time for a 3,000-m run field test against VO_2_ max scaled to body mass (kg) was estimated to be 0.485 (*p* = 0.003); **(C)** correlation coefficient of EPO for a 3,000-m run field test with VO_2_ max (L/min) was 0.757 (*p* <0.001). **(D)** correlation coefficient between EPO for a 3,000-m run field test and VO_2_ max (L/min) scaled to body mass was 0.349 (*p* = 0.04).

The results of interval validation for participants with body mass index <27.5 kg/m^2^ (N = 35) are revealed in Figure 4. The correlation coefficient of time for a 3,000-m run field test against VO_2_ max (L/min) was 0.453 (*p* = 0.006) (Figure 4A), and the correlation coefficient of time for a 3,000-m run field test with VO_2_ max scaled to body mass (kg) was 0.485 (*p* = 0.003) (Figure 4B). The correlation coefficient of EPO for a 3,000-m run field test with VO_2_ max (L/min) was 0.757 (*p* <0.001) (Figure 4C), and the correlation coefficient of EPO for a 3,000-m run field test against VO_2_ max scaled to body mass (kg) was 0.349 (*p* = 0.04) (Figure 4D). Although the correlation coefficients in the sample for interval validation were all lower than that in the overall sample (N = 45) receiving the CPET, all of the associations of time and EPO for a 3,000-m run with VO_2_ max in a CPET were significant, and the EPO association remained with the greatest strength.

### Estimation of VO_2_ max in CPET from time and EPO for a run field test

Based on the regression line in Figure 2B **,** formula 1, with regard to time for a run field test to estimate VO_2_ max scaled to body mass, can be derived as follows:

#### Formula 1

Y = −0.0386X + 67.151

X: Time for a 3,000-m field run (s)

Y: VO_2_ max scaled to body mass (mL/min/kg)

Based on the regression line in Figure 3A **,** formula 2, with regard to EPO for a run field test to estimate VO_2_ max, can be derived as follows:

#### Formula 2

Y = 3.7943X + 757.6

X: EPO for a 3000-m field run (watts)

Y: VO_2_ max (mL/min)

## Discussion

The principal finding of this study was that in young adults, the time for a run field test can be an acceptable estimate of VO_2_ max scaled to body mass obtained in CPET. In addition, the EPO for a run field test, calculated according to the kinetic energy theorem, can be a more precise estimate of VO_2_ max (L/min) than the time for a run field test.

Numerous studies have investigated the correlation between distance- or time-based run field test performance and VO_2_ max in CPET in adults (Mayorga-Vega et al., 2016). However, the correlation coefficients were distributed widely, ranging from 0.60 to 0.90, among various run field tests (Cooper, 1968). In addition, no consensus has been reached in previous studies to unify the VO_2_ max unit, with or without an adjustment for body mass, when analyzing the correlation. Furthermore, the correlation coefficients might vary even in the same run field test among different studies (Cooper, 1968; O’Gorman et al., 2000; Casajus and Castagna, 2007). For instance, Cooper found a high correlation between running distance and VO_2_ max scaled to body mass in a 12-min run field test in a sample of military males, in whom the correlation coefficient was estimated to be 0.897 (Cooper, 1968). In the O’Gorman et al. (2000) study, there was a moderate correlation in a 12-min run field test and in a 3,000-m run field test using a sample of physically fit young males, in whom the correlation coefficient was estimated equally to be 0.67 and −0.67. In contrast, the Casajus and Castagna study revealed a relatively lower correlation in a 12-min run field test in a sample of elite soccer players, in whom the correlation coefficient was estimated to be only 0.46 (Casajus and Castagna, 2007). In the present study, we demonstrated a moderate correlation of time for a 3,000-m run field test with VO_2_ max scaled to body mass, whereas the results showed a relatively lower correlation with VO_2_ max, which was not scaled to body mass. These findings are in line with previous studies and a meta-analysis (Serway and Jewett, 2004) made by Mayorga-Vega et al., which showed the correlation coefficient between time for a 3,000-m run field test and VO_2_ max scaled to a body mass of 12 studies, including 951 young adults, was estimated to be 0.70. We further highlighted the importance of the VO_2_ max unit for examining the correlation. It is reasonable that the examinees’ running velocity in run field tests was inversely related to their body mass. The correlation between the running velocity in a run field test and VO_2_ max scaled to body mass in CPET would theoretically result in the optimal result, except that the examinees had a similar level of body mass at baseline.

Some reports have shown a moderate-to-high correlation between peak cardiac power output (Watts) and VO_2_ max (L/min) not scaled to body mass in CPET in patients with heart failure, in whom the greatest correlation coefficient was 0.85, observed in those with recovering heart function (Jakovljevic et al., 2011), and a high correlation of peak power output with VO_2_ max in cycling athletes (correlation coefficient greater than 0.90) (Hawley and Noakes, 1992). The present study is the first report using EPO for a run field test to estimate VO_2_ max in CPET in young adults. Oxygen consumption generates energy, heat, and waste. Accordingly, it is apt to use peak exercise power output to assess VO_2_ max not adjusted for body mass in CPET for adults. In the present study, EPO was calculated according to the kinetic energy theorem and proportional to the square of the mean velocity in a 3,000-m run field test. The use of the square of the mean velocity was better than the mean velocity in the run field test to correlate with VO_2_ max in CPET in adults. This finding is consistent with a previous study on CPET, where peak power output was superior to the cycling speed to estimate VO_2_ max in athletes (Hawley and Noakes, 1992).

The present study, however, has a few limitations. First, the limited number of enrolled subjects is the major limitation to this preliminary study, and further study is required to expand the sample size to obtain greater power. Second, this study included only one woman and lacked multi-ethnic/racial diversity, making generalization of the results difficult. Third, although the annual military exercise test was held with some restrictions of the weather, there could have been a bias for differences in outdoor temperature and humidity, which may affect the running performance and EPO estimation. Fourth, as the study included merely healthy subjects, the results may not be appropriately applied to those with a mismatch between heart and lung functions. External validation should be performed in further study. Finally, since we chose mean running velocity in the kinetic formula, the maximum actual power output during the run test might be underestimated, possibly leading to a bias for the correlation with VO_2_ max.

## Conclusion

There have been no recommendations from the AHA regarding the role of time and EPO for a run field test to evaluate VO_2_ max in adults, and the VO_2_ max unit was not emphasized. Our findings suggest that in young adults, although the time for a distance run field test was an acceptable estimate of VO_2_ max scaled to body mass, EPO proportional to the square of the mean velocity in a run field test was found as a superior estimate of VO_2_ max than the time for a run field test in this population. Further studies are needed involving young women.

## Data Availability

The raw data supporting the conclusions of this article will be made available by the authors, without undue reservation.
