# Supplementary figures and images for: Estimated power output for a distance run and maximal oxygen uptake in young adults
Source: Front Physiol. 2023 Feb 7;14:1110802. doi: 10.3389/fphys.2023.1110802 (PMC9941527; doi:10.3389/fphys.2023.1110802)

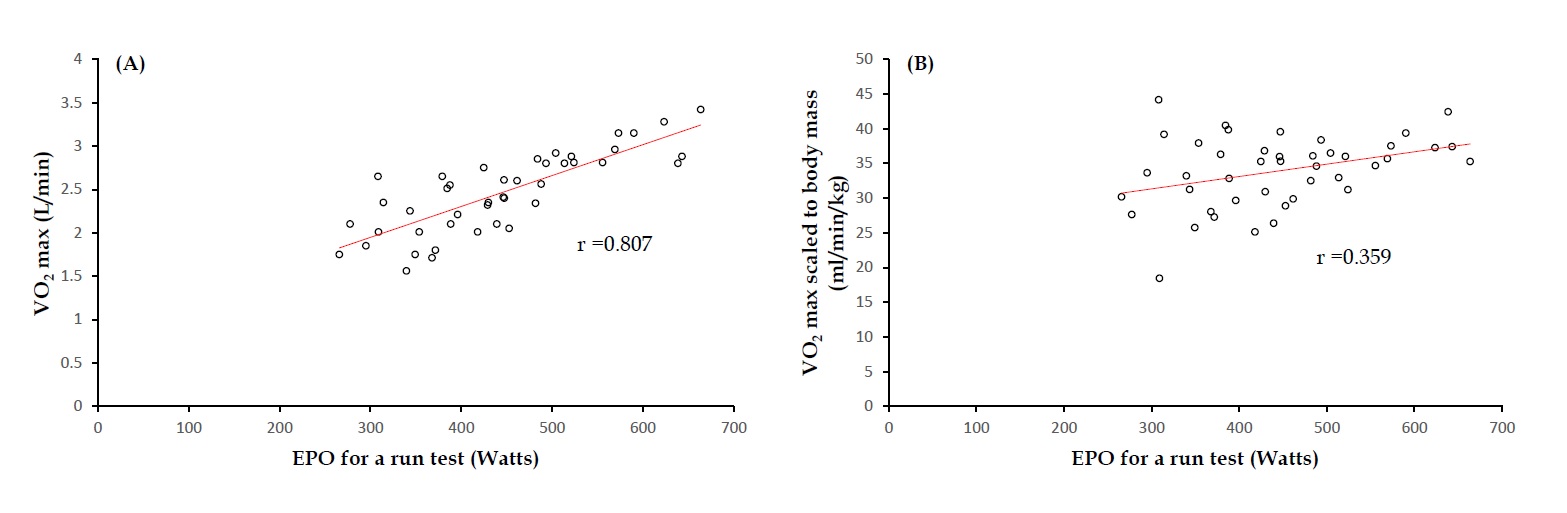

Supplement: Supplementary file 1 [file Image3.JPEG]

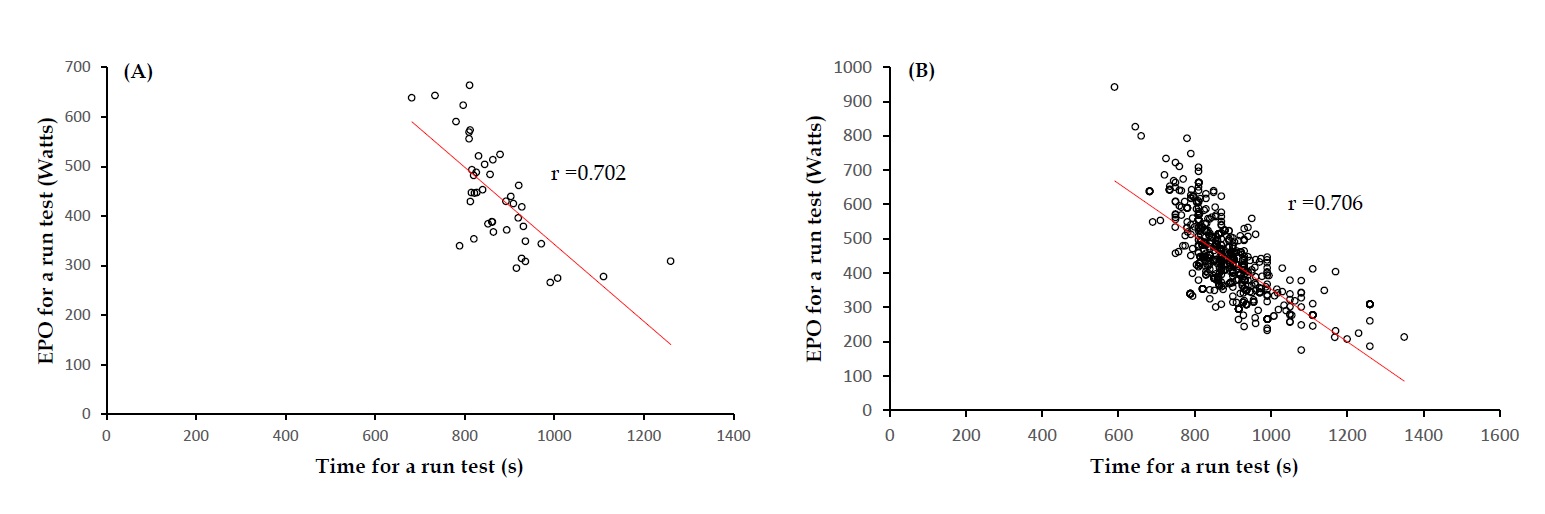

Supplement: Supplementary file 3 [file Image1.JPEG]

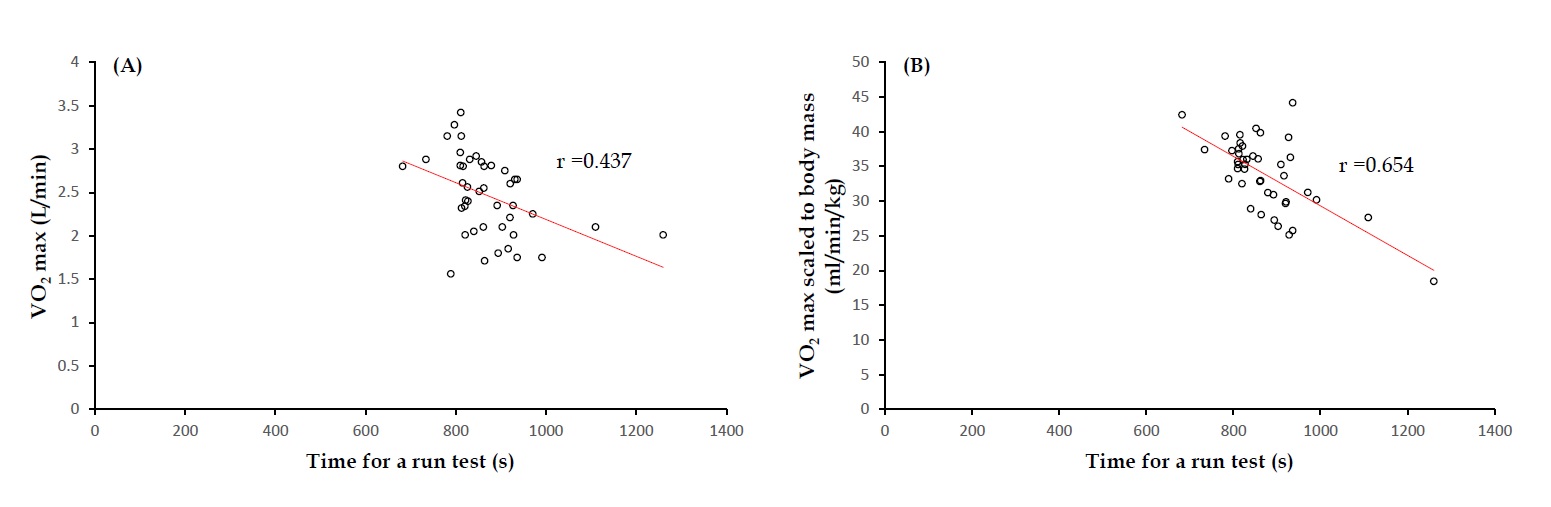

Supplement: Supplementary file 4 [file Image2.JPEG]
