# Supplementary material for: Estimated power output for a distance run and maximal oxygen uptake in young adults
Source: Front Physiol. 2023 Feb 7;14:1110802. doi: 10.3389/fphys.2023.1110802 (PMC9941527; doi:10.3389/fphys.2023.1110802)
Supplement: Supplementary file 2 [file Table1.docx]

**Supplemental Table 1. Clinical Characteristics of the Overall Male Participants for a Run Field Test and the Selected Male Participants for a Cardiopulmonary Exercise Testing**

|  | N =44 | N =911 | P-value |
| --- | --- | --- | --- |
| Sex, males (%) | 44 (100) | 911 (100) | - |
| Age, years | 30.12 ± 7.11 | 27.75 ± 5.98 | 0.01 |
| Body height, cm | 170.98 ± 6.41 | 172.03 ± 5.77 | 0.23 |
| Body mass, kg | 72.93 ± 12.00 | 73.47 ± 11.65 | 0.76 |
| Pulse rate, beats per min | 77.65 ± 11.40 | 67.17 ± 11.11 | <0.001 |
| Systolic blood pressure, mmHg | 128.28 ± 11.42 | 118.03 ± 12.65 | <0.001 |
| Diastolic blood pressure, mmHg | 80.34 ± 10.62 | 69.64 ± 9.85 | <0.001 |
| Time for a 3000-m run, secs | 874.84 ± 95.74 | 877.83 ± 94.17 | 0.83 |
| EPO for a 3000-m run, watts | 440.20 ± 105.99 | 440.36 ± 103.24 | 0.99 |

Abbreviation: EPO, estimated power output

EPO was defined as 1/2 x body mass (kg) x (3000-m/time for a 3000-m run test)^2^

**Supplemental Table 2. Clinical Characteristics of the Variables-Matched Participants for a Run Field Test and the Selected Participants for a Cardiopulmonary Exercise Testing**

|  | N =45 | N =707 | P-value |
| --- | --- | --- | --- |
| Sex, males (%) | 44 (97.8) | 882 (97.2) | 0.99 |
| Age, years | 29.93 ± 7.05 | 29.88 ± 6.62 | 0.96 |
| Body height, cm | 170.74 ± 6.47 | 171.32 ± 6.04 | 0.52 |
| Body mass, kg | 72.74 ± 11.81 | 74.97 ± 12.30 | 0.23 |
| Body mass index, kg/m^2^ | 24.95 ± 3.86 | 25.51 ± 3.81 | 0.34 |
| Pulse rate, beats per min | 77.50 ± 11.18 | 73.87 ± 12.29 | 0.06 |
| Systolic blood pressure, mmHg | 127.68 ± 11.83 | 128.42 ± 10.74 | 0.65 |
| Diastolic blood pressure, mmHg | 80.32 ± 10.39 | 79.97 ± 8.89 | 0.80 |
| Time for a 3000-m run, secs | 876.62 ± 94.34 | 884.51 ± 97.13 | 0.59 |
| EPO for a 3000-m run, watts | 437.44 ± 105.22 | 442.84 ± 106.15 | 0.74 |
| Moderate activity per week |  |  |  |
| 100-150 minutes | 8 (17.8) | 127 (18.0) | 0.95 |
| 150-300 minutes | 18 (40.0) | 268 (37.9) |  |
| >300 minutes | 19 (42.2) | 312 (44.1) |  |

Abbreviation: EPO, estimated power output

EPO was defined as 1/2 x body mass (kg) x (3000-m/time for a 3000-m run test)^2^
